# Task Design Influences Prosociality in Captive Chimpanzees (*Pan troglodytes*)

**DOI:** 10.1371/journal.pone.0103422

**Published:** 2014-09-05

**Authors:** Bailey R. House, Joan B. Silk, Susan P. Lambeth, Steven J. Schapiro

**Affiliations:** 1 Department of Anthropology, University of California Los Angeles, Los Angeles, California, United States of America; 2 School of Human Evolution & Social Change, Arizona State University, Tempe, Arizona, United States of America; 3 Michale E. Keeling Center for Comparative Medicine and Research, The University of Texas M. D. Anderson Cancer Center, Bastrop, Texas, United States of America; Brock University, Canada

## Abstract

Chimpanzees confer benefits on group members, both in the wild and in captive populations. Experimental studies of how animals allocate resources can provide useful insights about the motivations underlying prosocial behavior, and understanding the relationship between task design and prosocial behavior provides an important foundation for future research exploring these animals' social preferences. A number of studies have been designed to assess chimpanzees' preferences for outcomes that benefit others (prosocial preferences), but these studies vary greatly in both the results obtained and the methods used, and in most cases employ procedures that reduce critical features of naturalistic social interactions, such as partner choice. The focus of the current study is on understanding the link between experimental methodology and prosocial behavior in captive chimpanzees, rather than on describing these animals' social motivations themselves. We introduce a task design that avoids isolating subjects and allows them to freely decide whether to participate in the experiment. We explore key elements of the methods utilized in previous experiments in an effort to evaluate two possibilities that have been offered to explain why different experimental designs produce different results: (a) chimpanzees are less likely to deliver food to others when they obtain food for themselves, and (b) evidence of prosociality may be obscured by more “complex” experimental apparatuses (e.g., those including more components or alternative choices). Our results suggest that the complexity of laboratory tasks may generate observed variation in prosocial behavior in laboratory experiments, and highlights the need for more naturalistic research designs while also providing one example of such a paradigm.

## Introduction

The literature on social behavior in chimpanzees (*Pan troglodytes*) shows clearly that cooperation is common in these animals. They cooperate when patrolling territorial boundaries and attacking neighboring groups [1]; collaboratively hunting small prey [2]; sharing meat and other foods [3]–[5]; exchanging grooming for other valuable resources [6]–[8]; and jointly guarding mates [9]. However, this rich record of cooperation drawn from observational studies does not fully answer the question of how this behavior is motivated. Animals' social preferences lead them to select outcomes based on how they impact the relative payoffs of others, and different kinds of social preferences might be based on distinct motivations and guide prosocial behavior in different ways. For example, some prosocial behavior may be based on social preferences that positively value the welfare of others (e.g., prosocial preferences or unconditional altruism) while others may not (e.g., reciprocity, costly signaling). Studying naturalistic interactions is necessary to understand chimpanzee social behavior, but understanding the motivations behind this behavior and the specific social preferences at work requires studies that control parameters that are theoretically relevant to cooperative mechanisms and the evolutionary processes that would favor them, such as the relative benefits and costs that cooperative acts have for individuals and other group members. Experiments with captive animals offer opportunities to control these variables, and to describe animals' social motives.

Some experiments present captive chimpanzees with choices that have different material payoffs for themselves and others, and the choices animals make can reveal their underlying preferences. One such study allowed animals to select between two different payoff outcomes, both composed of fully visible food items [10]. One outcome (the “1/1” option) delivered a food reward to the animal making the selection (the Actor) and an identical reward to a familiar group member (the Recipient). The other outcome (“1/0”) delivered one reward to the Actor, but nothing to the Recipient. The selection between two payoff distributions like these is often referred to as the Prosocial Choice (PC) task, and Actors' behavior (and/or lack of behavior) can be grouped into three categories: (a) selecting the 1/1 outcome; (b) selecting the 1/0 outcome; or (c) doing nothing. The key measure in this experiment was whether subjects chose 1/1 more than 1/0. None of the chimpanzees from two different captive facilities differentiated between the Test trials (where a Recipient was present and could obtain benefits) and the Non-Social Control trials (where no Recipient was present), suggesting that the participant animals were indifferent to the welfare of conspecifics. These results were replicated in the same populations using a slightly different protocol [11], and in different populations [12], [13], all with procedures that used visible food rewards.

One exception to this pattern of results with the PC task is a recent study in which Actors were presented with a bucket containing many copies of two kinds of tokens, rather than an apparatus that delivered food items [14]. One kind of token could be traded with an experimenter to deliver a 1/1 outcome, while the other kind could be traded to deliver a 1/0 outcome. No food rewards were directly visible to Actors, as these rewards were hidden in an opaque wrapping. Actors selected the 1/1 outcome more frequently in the Test condition than in a Non-Social Control condition, implying that animals were prosocial in this study. The use of non-visible food rewards could be important, as the visibility of food rewards has previously been shown to substantially impact animals' choices in experimental tasks [15]. Alternatively, the lack of an apparatus and a simultaneous presentation of binary choices could be construed as reducing the complexity of this task relative to prior PC tasks.

Other kinds of experimental designs have led to different conclusions about chimpanzee prosocial behavior. In a body of experiments called Instrumental Helping (IH) tasks Actors' behavior (and/or lack of behavior) can be grouped into two categories: (a) assist a conspecific or (b) do nothing. As these studies lack a simultaneous presentation of binary choices, they could also be construed as less complex than prior PC tasks. In IH studies chimpanzees seem to be sensitive to outcomes obtained by others because they provide help to Recipients in a variety of different ways: unlocking a door that was obstructing access to food rewards [16], releasing food rewards so they slide within reach of a Recipient [17], pulling a handle to help a Recipient move a food reward within reach [18], or transferring a tool to a partner who needs it to obtain a food reward [19], [20]. In these studies Actors do not obtain food rewards for themselves when they provide help to a conspecific, and it is possible that this explains why prosociality is observed in IH tasks but not PC tasks [21]. Recent research suggests that the mere presence of desirable food does not influence subject animals' willingness to provide help [17], [22], but Melis et al. [17] noted that animals may be less influenced by the mere presence of food rewards than they are by obtaining food for themselves at the same time as they deliver food to conspecifics. However, there is also evidence that animals are no more prosocial when they can deliver food to conspecifics *after* they have already obtained food for themselves [11]. The results of IH studies have been taken to reveal social preferences in chimpanzees that positively value the payoffs obtained by others.

In a third body of experiments referred to as Inequity Aversion (IA) studies, chimpanzees may refuse to participate in a task if they receive a lower payoff for their participation than a conspecific partner, and it has been argued that such behavior is evidence for an aversion to inequity [23]. Inequity aversion might stabilize cooperation by incentivizing individuals to reject inequitable offers made by their partners in favor of other alternatives [24], [25]. If chimpanzees are averse to inequity then this points to preferences that are sensitive to payoffs obtained by others. However, one study was unable to replicate results using similar procedures [26], and controversy remains both over how to interpret this phenomenon [27] and the specific methods and contexts necessary for eliciting an aversion to inequity in chimpanzees [28], [29]. Results from a different paradigm suggest that captive chimpanzees use an apparatus to discard food items that they themselves cannot access, but whether or not they discard this food isn't affected by whether or not another animal is feeding on that food [30]. This suggests that these animals weren't particularly motivated to prevent other animals from obtaining more rewards than themselves.

Differences in methodology, task demands, and the rewards animals obtain across these bodies of research make it very difficult to reconcile divergent findings about chimpanzee sociality. PC tasks typically allow animals to obtain food rewards for themselves while delivering food to others, while in IH tasks Actors do not obtain food at the same time as do Recipients. The mere presence of food does not inhibit subjects' prosocial behavior [17], but it is possible that seeing rewards that animals can obtain for themselves obscures their prosocial tendencies. This might also explain why one PC study using tokens and non-visible rewards found evidence of prosociality [14] where other PC studies did not. A second difference is that PC tasks present animals with a larger number of discrete choices than do IH tasks. The complexity of the tasks (e.g., the number of outcomes to choose from, the number of locations or events to keep track of, etc.) may affect the chimpanzees' performance. This could be due to more complex tasks being too cognitively demanding for participant animals, but it could also be due to more complex laboratory tasks being less ecologically relevant or less interesting to chimpanzees, rather than them being too difficult to understand.

Here, we explore prosocial behavior in captive chimpanzees using a procedure that manipulates payoff outcomes for Actors and Recipients in ways similar to the PC, IH, and IA suites of tasks. We investigate whether chimpanzees are less prosocial when (a) tasks are more complex and (b) when animals obtain rewards for themselves, two hypotheses that may explain differences in observed levels of prosocial behavior across studies. No prior study has used a common research design to explore and compare findings from the PC, IH, and IA paradigms. Previous studies have explored whether the mere presence of food rewards reduces chimpanzee prosociality [17], but found no evidence that this underlies the differences in results from different research methods. We expand on this work by exploring whether animals are less prosocial when they concurrently obtain food rewards for themselves, in a manner that bridges prior work by Melis et al. [17] and Jensen et al. [12]. Study 1 presents captive chimpanzees with a prosocial task comparable to IH tasks, and tests whether animals prefer outcomes that benefit others. We also test whether animals are averse to prosocial outcomes that result in disadvantageous inequity. Study 2 presents chimpanzees with a more-complex prosocial task that is more analogous to previous PC tasks. In both studies, we manipulate whether Actors obtain payoffs for themselves at the same time that they deliver payoffs to Recipients.

We also designed our tasks to be more naturalistic by testing animals with unconstrained access to their social group, allowing them substantial freedom of choice over when to participate in the experimental trials and with which group members. Other studies have taken a similar approach by also testing animals within their home social groups [31]–[33]. There is substantial evidence that partner choice is an important component of chimpanzee social interactions, both in the wild [3], [6]–[8], [34], [35] and in laboratory experiments [36]. Allowing animals complete freedom of choice over when to participate and with whom creates a more naturalistic social interaction than typically allowed when animals are isolated from their social groups. Such opportunities for partner choice could be more likely to elicit prosocial behavior, given prior evidence that animals engage in partner choice in similar tasks [36], and designs such as the one introduced here can be used in the future to study the role of reciprocity in chimpanzee social behavior over longer time periods.

We highlight that our focus is primarily on prosocial behavior in captive chimpanzees, rather than on describing these animals' social preferences or prosocial motivations. Social preferences such as reciprocity, prosocial preferences, or an aversion to inequity are cognitive mechanisms that guide animals' patterns of prosocial behavior. Here we focus on describing the behavior itself, how it can be elicited in relatively more naturalistic social interactions, and how it varies across different kinds of task designs. Understanding the relationship between task designs and prosocial behavior will facilitate future research exploring the social preferences behind chimpanzee prosociality, and also research seeking to bridge the gap between studies of these animals in the wild and in the laboratory.

## Study 1

**Figure 1:**
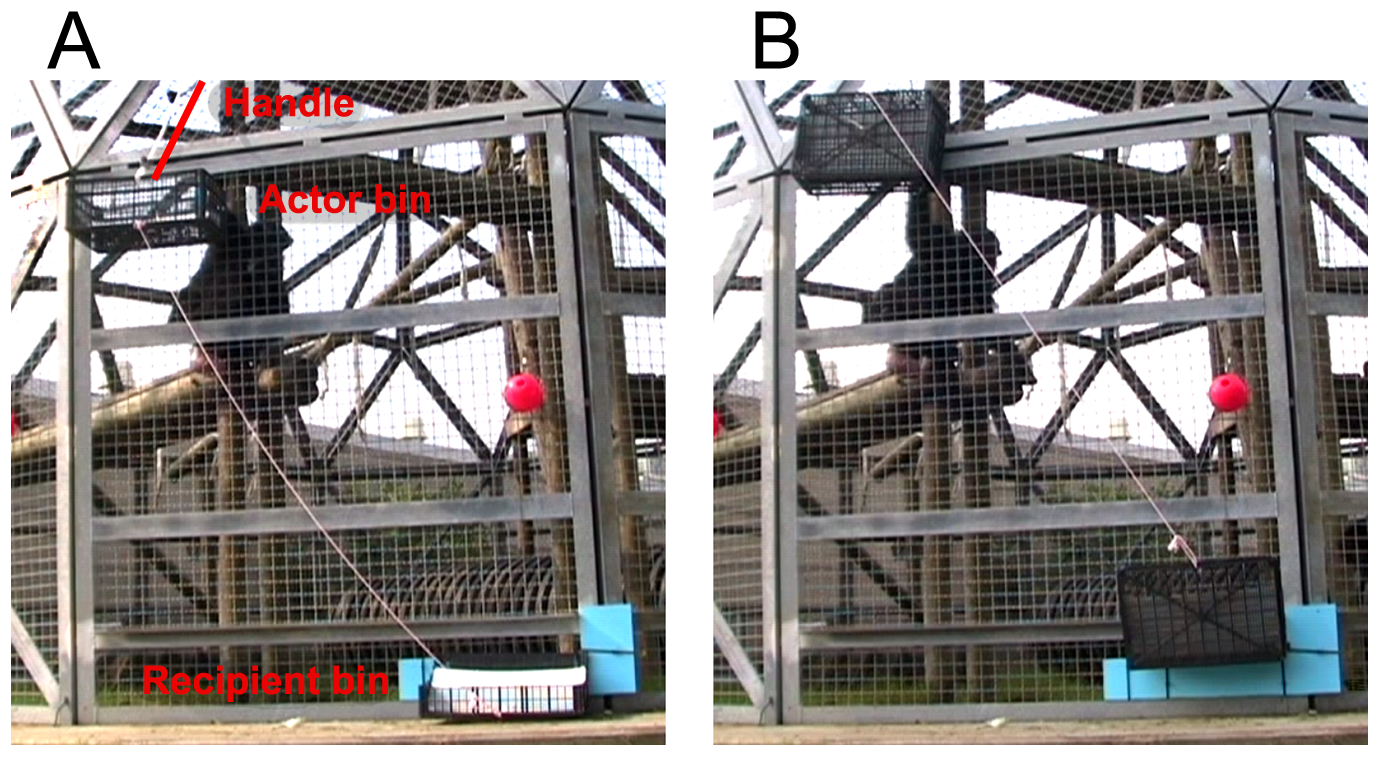
Apparatus used in Study 1. (A): The apparatus in the No Access position, with bins at rest where animals could not obtain any food. (B): The apparatus in the Access position, with bins pivoted upward so that food rewards were in reach of animals inside the enclosure. Pulling the handle moved the apparatus from No Access (A) to Access (B). While pulling the handle, the Actor could acquire food placed in the Actor bin, but could not reach food in the Recipient bin. If the Actor released the handle both bins returned immediately to the No Access position and any food remaining in the Recipient bin rolled back out of reach.

Subjects were socially housed chimpanzees at the Michale E. Keeling Center for Comparative Medicine & Research (KCCMR) in Bastrop, TX. Actor animals were able to deliver payoffs (pieces of apple) to themselves and to other group members by using the apparatus depicted in Figure 1. Whether payoffs could be delivered to Actors, to Recipients, to both, or to neither was varied across trials. This apparatus consisted of two plastic food bins (the Actor bin and Recipient bin) that were anchored to the enclosure more than a full arm span apart, a distance which ensured that the Actor could obtain rewards directly from the Actor bin, but not directly from the Recipient bin. Only one animal at a time could act as Actor and operate the apparatus. The apparatus was novel to all participants, but was similar to a familiar enrichment device and its mechanics were thus familiar to the participant animals. Trials were video recorded. All animals in the group had free access to the apparatus, and individuals thus varied substantially in the number of observations they contributed to the dataset (ranging from 1 to 124 observations; median: 31; see Table S1 for details, and demographic data on participants). During testing the number of animals near the apparatus varied and some animals participated more than others, with less-dominant members of each social group not gaining as much access to the apparatus as more-dominant group members. However, dominant individuals did not exclusively monopolize the apparatus (i.e., the Actor and Recipient roles weren't always filled by the same individuals), and animals with Medium dominance had the most access (see Table S1). There were often multiple possible Recipients on a given trial, as animals clustered around the Recipient bin (see below for details about the apparatus).

Payoffs for Actors and Recipients varied across trials (Table 1). If Actors were prosocial they should have pulled more frequently on trials where the Recipient obtained a reward (0/1) than on trials where the Recipient obtained nothing (0/0), and also more than on trials where the Recipient obtained nothing but a food item was still visible (0/0(1); see Table 1). Recipients were able to see whether or not food was placed in the Actor bin, and food placed in the Recipient bin was always fully visible to the Actor. For the 0/0(1) payoff distribution, the food item was placed on the ground next to the Recipient bin, no more than 12 inches from where the food was located when it was placed inside the Recipient bin (as in the 0/1 payoff distribution). The food in the 0/0(1) condition was just as visible to the Actor as was the food in the 0/1 condition. 0/1 most closely conforms to the payoff outcome used in IH tasks, with only Recipients obtaining a benefit at a very low cost to Actors (i.e., the energetic cost of pulling the handle). Study 1 differs from prior IH studies in several methodological respects, but it also shares important features with IH studies where one animal was able to pull a handle to open a door for a Recipient [16], or where one animal was able to remove a pin to make food move within reach of a Recipient [17].

**Table 1:** Payoff distributions used in Study 1.

Payoff distributions	Payoff for Actor	Payoff for Recipient
0/0	Zero	Zero
0/1	Zero	One reward
1/0	One reward	Zero
1/1	One reward	One reward
1/3	One reward	Three rewards
0/0 (1)	Zero	Zero *(one food item placed on the ground next to the Recipient's bin)*

We label these distributions using the convention of “(Actor's payoff)/(Recipient's payoff).”

As long as Actors in the current study desired the food payoffs then they would be expected to pull the handle at near-ceiling levels when they themselves obtained rewards (1/0 and 1/1), regardless of their social preferences. However, if chimpanzees were averse to disadvantageous inequity then Actors would be expected to pull the handle less when Recipients obtained a greater payoff than they themselves did (1/3).

### Results

The outcome variable was the binary parameter *Actor Pulled Handle*, which captured whether the Actor pulled the handle (coded as “1”) or did not pull (coded as “0”). Whether or not the Actor pulled the handle was coded live, and a second coding assessed the reliability of the live coding using 40 trials recorded with video. The second coding agreed with the live coding on every trial (Kappa = 1.00).

**Figure 2:**
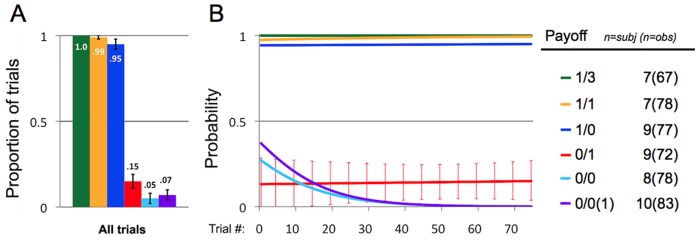
Data from Study 1. (1A) Proportions of all trials during which actors pulled the handle to operate the apparatus, for each of payoff distribution. Error bars reflect one SE of the mean but do not control for non-independence, though models suggest there is little between-subjects behavioral variation (see Table S2). Numbers of subjects and observations are listed on the right. (1B) Logistic functions modeling the effect of *Actor's Trial Number* on the probability that Actors will pull the handle (see Table S4 for regression model estimates). These probabilities are comparable to the proportions from Figure 1A. Y-axis represents the probability that actors will pull the handle. X-axis represents the number of trials that an Actor has received previously (across all payoff distributions). Error bars around the red line (modeling the probability of pulling the handle for 0/1 trials) are estimated 95% confidence intervals, and control for non-independence.

We included Actor identity as a random effect in our regression models to control for non-independence of observations. *Actor's Trial Number* codes for the total number of test trials that Actors had previously participated in (across all six payoff distributions). Figure 2A illustrates that animals pulled the handle at different rates across the six payoff distributions, and Actors pulled the handle much more frequently when food was placed in the Actor bin (1/3, 1/1, 1/0) relative to when no food was placed in the Actor bin (0/1, 0/0, 0/0(1)). However, though the overall likelihood of pulling the handle was low when there was no food in the Actor bin, Actors were two to three times as likely to pull when Recipients received rewards (0/1: mean number of trials/SD = .15/.36, N = 72) relative to when Recipients did not receive rewards (0/0: mean/SD = .05/.22, N = 78; and 0/0(1): mean/SD = .07/.26, N = 83).

Recipients usually obtained available food when the Actor pulled the handle on trials where food was placed in the Recipient's bin. Recipients failed to obtain all food available to them on only two trials (2% of total) for the 0/1 payoff distribution, and four trials (5% of total) for the 1/1 payoff distribution. For the 1/3 payoff distribution Recipients failed to obtain all of the food rewards on 21 of the trials (30%), but no instances of aggression by Recipients toward Actors were observed.

Multilevel (generalized mixed effects) logistic regression analyses were performed in STATA 11 (using the xtlogit function). Our regression model (see Table S2) uses 0/0 as a reference point and asks whether Actors pull the handle more or less frequently for the five other payoff distributions than they do for 0/0. The coefficient for 0/0(1) is positive but smaller than its standard error (coefficient/SE = .34/.67), suggesting that animals were not substantially more likely to pull the handle when a food item was present but inaccessible. The coefficient for 0/1 is positive and about twice as large as its standard error (coef./SE = 1.22/.61), indicating that Actors were more likely to pull the handle when a food item was present *and* placed inside the Recipient bin. The coefficient for 0/1 (Table S2) translates to an Odds Ratio of 3.38 (SE: 2.07), indicating that animals were 3.38 times more likely to pull the handle for 0/1 (15% of trials) than they were for 0/0 (5% of trials). See Table S3 for an analysis showing how this result is robust to dropping individual participant animals from the sample. The coefficients were all positive and much larger than their standard errors for 1/0 (coef./SE = 5.96/.77) and 1/1 (coef./SE = 7.42/1.16), and Actors pulled the handle on every 1/3 trial. This indicates that Actors were very likely to pull when they would receive food. The random effect parameter reflects the variation in the outcome measure across individuals. Its coefficient is small, and also smaller than its SE, suggesting that subjects were largely similar in their behavior (coef./SE = .30/.41; see Table S2). *Actor's Trial Number* predicted animals' behavior to different degrees across trial types (Figure 2B, see Table S4 for models). For 1/1, 1/0, 1/3, and 0/1, the Actor's likelihood of pulling the handle changed little as trial number increased. For 0/0 and 0/0(1)—the two trial types in which neither Actor nor Recipient ever obtained payoffs—the predicted probability of pulling for 0/0 and 0/0(1) drops substantially as a function of experience with more trials (see Figure 2B, and Table S4).

**Figure 3:**
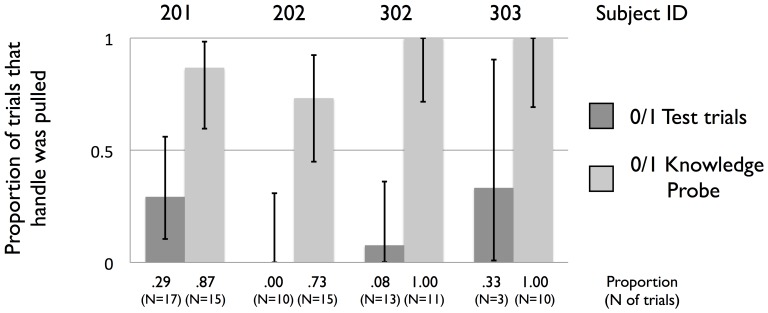
Data from Knowledge Probe (Study 1). Proportions of trials during which Actors pulled the handle to operate the apparatus during the Test trials (dark grey bars) and during the Knowledge Probe (light grey bars), for the 0/1 payoff distribution only. Each pair of bars corresponds to one of the Actors who participated in the Knowledge Probe. Values below the x-axis provide means, standard deviations, and sample sizes. Error bars reflect 95% confidence intervals.

In the Knowledge Probe we explored whether Actors were more likely to pull the handle when the apparatus was modified to allow them to directly access food from the Recipient bin (four animals were willing to be isolated for this test; see Methods section at the end for details). The purpose of the Knowledge Probe was to confirm that animals' more frequent pulling of the 0/1 outcome (relative to the 0/0 outcome) was due to an understanding that doing so allowed access to food placed in the Recipient bin. We found that in the Knowledge Probe animals were indeed much more likely to pull the handle for 0/1 than they had been in the Test trials (OR = 44.6, see Table S5 for regression model; see Figure 3 for means, standard deviations, and sample sizes), and for most of the animals they showed this pattern from the very beginning of the Knowledge Probe (see Figure S1, Table S6 and Table S7). For 1/1, animals pulled the handle on every trial in both the Knowledge Probe and Test trials, and for 0/0 Actor animals pulled the handle at comparably low rates in both the Knowledge Probe and Test trials (see Figure S2).

### Discussion

Actors were far more likely to pull the handle on trials in which food was in the Actor bin. On those trials in which no food was placed in the Actor bin, Actors were also relatively more likely to pull the handle on trials in which food was placed in the Recipient bin. Animals demonstrated their comprehension of the task by showing different responses to the different trial types. With experience, Actors remained consistent in their tendency to pull the handle when Recipients obtained a benefit (0/1), while simultaneously becoming less likely to pull the handle when neither Actor nor Recipient benefitted (0/0 and 0/0(1)). As all three of these conditions have the same direct payoff for the Actor, animals would only show this pattern is if they were attending to the rewards in the Recipient bin, and if they understood that pulling the handle made rewards available in the 0/1 condition (but not the other two conditions). This pattern thus shows that Actors understood that pulling the handle made food in the Recipient bin available. Given that only Recipients could directly access food in the Recipient bin, the pattern of results suggests that Actors comprehended how their choices impacted group members, and also that they learned to ignore trials that didn't deliver benefits to conspecifics or to themselves. Additionally, Actors were more willing to pull the handle in the Knowledge Probe than in the Test trials, but only when food was placed only in the Recipient bin (0/1). This again shows that they understood that pulling the handle caused food in the Recipient bin to become available. These results are independent, as each could be true while the others could have been false, and each is consistent with the fact that animals understood the critical feature of the device: pulling the handle made food in the Recipient's bin available.

One possibility is that Actors pulled the handle because they wished to make food accessible to the interior of the enclosure, but not specifically because they wished for Recipients to obtain it. This is possible, particularly because there was always a non-zero probability that the Actor would receive food once it was retrieved by a Recipient. However, given that only Recipients could directly access the food in the Recipient's bin (and Actors were relatively far away), the likelihood was always that the food would be consumed before the Actor got to it. Thus, the overall pattern of results strongly points to Actors understanding that pulling the handle was likely to deliver food that only another group member would consume. Overall, Study 1 suggests that captive chimpanzee Actors display behavior that confers low rates of prosocial outcomes on Recipients within a task similar to the one used in IH tasks. Again, the motives behind animals' behavior is not obvious, but nonetheless Actor's in Study 1 did show a small tendency to act in a way that was more likely to confer benefits on others than it was on themselves.

In Study 2 we explore whether chimpanzees also behave prosocially when we modify the same apparatus to create a situation that more closely resembles the PC task [10], [13], [37]. We also explore whether chimpanzees will be more prosocial in the PC task if they do not obtain food for themselves concurrently with delivering food to conspecifics, as proposed by Melis et al. [17]. Jensen et al. [12] previously tested this hypothesis and found no support for it, but using a different method than Melis et al. [17]. Here we extend these findings by exploring whether we can obtain more evidence of prosociality in chimpanzees than did Jensen et al. [12] by using our apparatus from Study 1, which (like the apparatus used by Melis et al. [17]) elicits non-zero rates of helping behavior.

## Study 2

**Figure 4:**
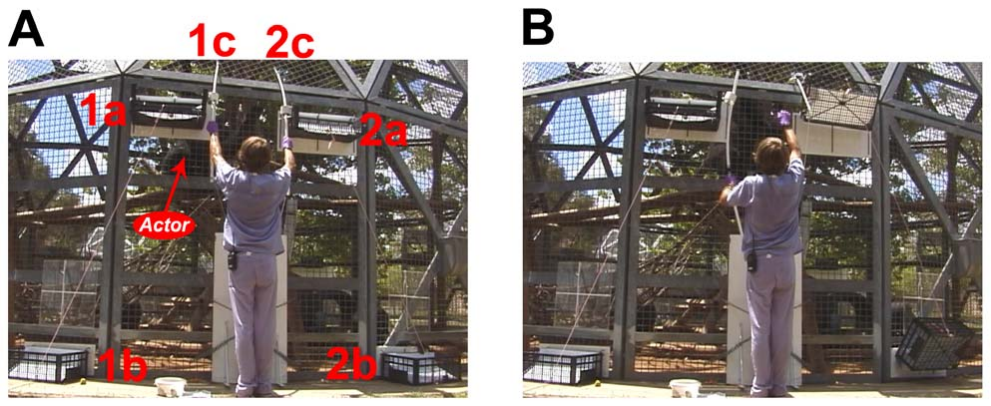
Apparatus used in Study 2. (A): The apparatus in the No Access position, before the Actor has pulled either of the two handles. (B): The apparatus in the Access position, after the right handle has been pulled by the Actor, which retracts the left handle so that it becomes inaccessible (i.e., it is now impossible for the Actor to move the left Actor bin and left Recipient bin into the Access position). (1a): left Actor bin. (2a): right Actor bin. (1b): left Recipient bin. (2b): right Recipient bin. (1c): left handle. (2c): right handle.

Participants were drawn from three social groups at KCCMR, each containing 4–7 animals. None of these groups were included in Study 1, and all participants were naïve to the apparatus in Study 2. Once again, trials were video recorded. During trials, Actors were faced with a choice between two of the same 2-bin mechanisms used in Study 1, arranged side by side (see Figure 4). When an Actor pulled the handle that operated one of the mechanisms the device automatically retracted the other mechanism's handle (i.e., the experimenter did not retract the handle). This prevented Actors from operating more than one mechanism within a single trial, and if both handles were pulled then neither apparatus delivered payoffs until one handle was released and fully retracted. All animals in the social group again had free access to the apparatus, and there was again substantial variation in the number of observations that the Actors contributed to the dataset (ranging from 23 to 332 observations; median: 141; see Table S8 for details, and demographic data on participants). Dominant individuals again likely had greater access to the apparatus, but did not monopolize it.

Payoffs for Actors and Recipients (pieces of banana) again varied across trials (Table 2). If subjects preferred 1/1 over 1/0 (the *1/1 v. 1/0* condition), but not 1/1 over 1/0(1) (the *1/1 v. 1/0(1)* condition), this would suggest that they were simply biased by the greater quantity of food associated with 1/1. The *0/1 v. 0/0* condition was equivalent to the *1/1 v. 1/0* condition except Actors obtained no food rewards for themselves regardless of which option they selected. The *1/1 v. 0/1* condition evaluated animals' attentiveness to the task by providing them with choices that only varied in the payoffs they conferred on Actors themselves. If animals pulled each handle only half the time in the *1/1 v. 0/1* condition, this would indicate that they did not understand or were not attending to the apparatus, and were merely selecting handles at random.

**Table 2:** Payoffs distributions used in Study 2.

Payoff Distribution	Description
1/1 v. 1/0	Prosocial outcome vs. Non-Prosocial outcome. Rewards for actor.
1/1 v. 1/0 (1)	Prosocial outcome vs. Non-Prosocial outcome. Rewards for actor. *(one food item placed on the ground next to the Recipient's bin for the 1/0 payoff.)*
0/1 v. 0/0	Prosocial outcome vs. Non-Prosocial outcome. No rewards for actor.
1/1 v. 0/1	Payoff for self vs. No payoff for self.

We label these distributions using the convention of “(Actor's payoff)/(Recipient's payoff).”

### Results

The primary outcome variable was the binary parameter *Actor Chose Prosocial Outcome*. For the *1/1 v. 1/0*, *1/1 v. 1/0(1)*, and *0/1 v. 0/0* conditions, prosocial choices (1/1, 1/1, and 0/1, repectively) were coded as “1”, and non-prosocial choices (1/0, 1/0(1), and 0/0) were coded as “0”. For the *1/1 v. 0/1* condition, 1/1 was coded as “1” and choices of 0/1 as “0”. Our primary analyses exclude trials in which Actors selected neither of the payoff distributions, and we separately analyze trials wherein Actors make no choice (i.e., “do nothing”). Multi-level logistic regressions (generalized mixed effects models, STATA 11 function “xtlogit”) controlled for non-independence of observations by considering Actor identity as a random effect, and we investigated how *Actor's Trial Number* predicted animals' behavior in each of the four conditions.

Animals behaved differently across the conditions. When we examine all trials together, the mean rate at which Actors selected the prosocial outcome was .36 (SE = .03) for the *0/1 v. 0/0* condition, .54 (SE = .03) for the *1/1 v. 1/0* condition, .50 (SE = .03) for the *1/1 v. 1/0(1)* condition, and .98 (SE = .01) for the *1/1 v. 0/1* condition. This includes trials during which animals selected one of the two handles, and also trials during which Actors did nothing (i.e., chose neither handle). Actors selected the prosocial outcome nearly 50% of the time for the *1/1 v. 1/0* and *1/1 v. 1/0(1)* conditions, but below 50% of the time for the *0/1 v. 0/0* condition, and well above 50% of the time for the *1/1 v. 0/1* condition. Overall rates of 0/1 choices (i.e., prosocial choices) in the *0/1 v. 0/0* condition of Study 2 (mean = .36) are thus higher than were rates of 0/1 choices in Study 1 (mean = .15). However, rates of 0/0 choices were also higher in the *0/1 v. 0/0* condition of Study 2 (mean = .27) than they were in Study 1 (mean = .05), and Actors also frequently chose nether of the outcomes during trials (mean = .38). This pattern suggests that animals were not strongly biased towards the prosocial outcome in the *0/1 v. 0/0* condition (i.e., 0/1). However, it does appear to be true that Actors were more willing to “do nothing” in Study 1 than in Study 2, at least when they obtained no rewards for themselves.

**Figure 5:**
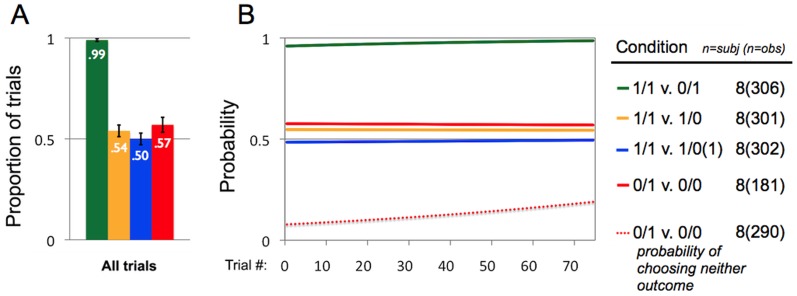
Data from Study 2. (3A) Proportion of trials during which actors selected the prosocial outcome (for *1/1 v. 1/0*, *1/1 v. 1/0(1)*, and *0/1 v. 0/0* conditions) or the self-maximizing outcome (for *1/1 v. 0/1*), on trials where the Actor selected one of the two options. If Actors were indifferent to the payoffs obtained by recipients, they would be expected to pull the handle on half of the trials (proportion of .50). Numbers of subjects and observations are listed on the right. (3B) Logistic regressions representing the probability of actors' choices as a function of *Actor's Trial Number* (see Table S9 for regression model estimates). Solid lines depict the estimated probabilities that actors will select the prosocial or self-maximizing outcome, which are comparable to the proportions from Figure 3A. Y-axis represents the probability that actors will select the prosocial or self-maximizing outcome, and the x-axis represents the number of trials that an actor has received previously (across all payoff distributions/conditions). The red dotted line depicts the estimated probability that actors will do nothing (i.e., pull neither of the handles) in the *0/1 v. 0/0* condition (see Table S10 for regression model estimates).

Actors' rates of “doing nothing” were much higher in the *0/1 v. 0/0* condition (mean/SE = .38/.03) than in the *1/1 v. 1/0* condition (.01/.005), *1/1 v. 1/0(1)* condition (.003/.003), and *1/1 v. 0/1* condition (.01/.005). When we focus only on trials in which Actors chose one of the two handles, animals only displayed a systematic bias in their selection of one mechanism over the other in the *1/1 v. 0/1* condition, when their own rewards were the only payoffs at stake (Figure 5A). For trials in which only the Recipient's payoffs were at stake, Actors chose the prosocial outcome slightly more than 50% of the time for the conditions *1/1 v. 1/0* (mean/SE = .57/.04) and *0/1 v. 0/0* (.54/.03), but exactly 50% of the time for the *1/1 v. 1/0(1)* condition (.50/.03). In the *1/1 v. 0/1* condition animals chose the outcome that delivered a reward to themselves on nearly every trial (mean/SE = .99/.01). Across conditions, the estimated probability that Actors chose the prosocial outcome was very stable as trial number increased, and the random effect parameter was small, suggesting that there was little behavioral variation across subjects (Figure 5B, see Table S9 for regression models). However, increasing trial number did lead to a greater probability that Actors would do nothing (i.e. pull neither of the handles) in the *0/1 v. 0/0* condition (Figure 5B, red dotted line), but not in any of the other conditions (see Table S10 for regression models). These results suggest that in the *0/1 v. 0/0* condition animals were more likely to pull neither handle than they were in the other conditions, and that this pattern increased as the experiment progressed.

### Discussion

Our prior results from Study 1 suggest that chimpanzees from this population understood how to use these apparatuses to make food accessible to Recipients, and the results from Study 2 suggest that chimpanzees understood the how to use these apparatuses to systematically deliver food to themselves (in the *1/1 v. 0/1* condition). Overall, animals were very slightly more likely to select the prosocial outcome over the non-prosocial outcome in both the *1/1 v. 1/0* and *0/1 v. 0/0* conditions, and rates of selecting the prosocial outcome did not differ across these two conditions, indicating that Actors were not more prosocial when they did not obtain rewards for themselves. However, Actors were not more likely to select the prosocial outcome over the non-prosocial outcome in the *1/1 v. 1/0(1)* condition. This implies that in the *1/1 v. 1/0* and *0/1 v. 0/0* conditions animals were not biased toward the prosocial outcome per se, but they were biased toward the location containing greater quantities of food, a phenomenon that has been reported previously in chimpanzees [10], [15], [38]. Given Actors' systematic behavior in the *1/1 v. 0/1* condition, these results suggest that animals were not merely inattentive to the apparatus, nor were they simply selecting handles at random. Instead, these results are consistent with animals being indifferent to the payoffs obtained by Recipients in Study 2.

## General Discussion

We observed that captive chimpanzees acted to confer benefits on conspecifics in some laboratory contexts (though at low rates), and our findings suggest that methodological differences may help explain discontinuities in results across studies. In this way these data are consistent with studies of wild animals, and with the results of laboratory studies showing prosocial behavior in IH studies [16]–[19] and one PC study [14]. However, it is crucial that empirical studies distinguish how their results pertain to prosocial behavior and to prosocial motivations. Data from these kinds of laboratory studies (and also naturalistic interactions) cannot reveal the nature of chimpanzees' social preferences. This is because these data describe animals' behavior but do not disambiguate various possible preferences or social motivations, of which there are several possibilities in the current studies. Animals might act prosocial for motives that do not positively weight others' welfare (e.g., trying to elicit reciprocity or to avoid conflict, signaling one's intrinsic quality as a mate), and so studying whether chimpanzees confer prosocial outcomes says more about behavior than motivations. This problem has been highlighted recently by Heyes [39], who points out that prior evidence for prosocial behavior [14] could be a product of conditioning—not a psychology motivated to deliver benefits to conspecifics. Chimpanzees may be adaptively prosocial even if they lack psychological mechanisms that value the welfare of others independently of their own welfare. This may be true in the current study as well as all previous studies in the literature. However, we emphasize that the psychological motives underlying prosocial behavior in chimpanzees can only be studied after first developing methods that consistently elicit it. These methods will be critical not just for integrating studies of chimpanzee sociality from the wild and the laboratory, but also for making phylogenetic comparisons between studies of prosociality in chimpanzees and those in other ape species [40], and also cercopithecines [41], [42] and platyrrhines [43], [44].

Although chimpanzees acted prosocially in Study 1 the rates of animals' prosocial choices were quite modest, which starkly contrasts with the prosocial acts frequently observed in laboratory interactions between humans [45]–[48]. It is thus important to consider the different motives that could have generated the results. In Study 1 Actors were only 10% more likely to pull the handle on 0/1 trials (15%) than 0/0 trials (5%), implying that Actors were weakly influenced by the payoffs obtained by Recipients in this task. Harassment or begging by Recipients could motivate this kind of behavior [5], though there were no observed instances begging or aggression directed by Recipients towards Actors. Alternatively, this behavior could be motivated by reciprocity given that our subject animals were permitted unconstrained partner choice in non-anonymous interactions with long-term social partners. There is evidence for reciprocity in captive chimpanzees [49], but analyses of reciprocity in the current paradigm would require additional data, as free partner choice resulted in particular pairs of Actors and Recipients being disproportionately represented in the dataset, reducing the necessary variation in Actor/Recipient pairings. Evidence from studies of wild chimpanzees suggest that there may be more evidence for long-term reciprocal exchanges [50], [51] than for short-term exchanges [51], [52], and future work on reciprocity in captive populations should also explore reciprocity over the long-term [53].

Our results are not consistent with the hypothesis that chimpanzees are less prosocial when they obtain rewards for themselves [17], [22], because in Study 2 Actors were equally indifferent to the payoffs obtained by others in both the *0/1 v. 0/0* and the *1/1 v. 1/0* conditions. This replicates similar findings by Jensen et al. [12] showing that animals were not more prosocial in a condition where they did not obtain rewards for themselves, but we do so using an experimental apparatus that did elicit low levels of prosocial behavior in Study 1. Our findings revealing evidence for prosociality in Study 1 but not in Study 2 suggest that more complicated experimental tasks involving choices between more possible outcomes (such as the one used in Study 2) may be less likely to elicit prosocial behavior in captive chimpanzees relative to simpler tasks involving choices between fewer possible outcomes (such as the one used in Study 1). In Study 1, animals showed a weak tendency to deliver prosocial outcomes when presented with a single apparatus that they could either use or not use (similar to IH tasks), but animals showed no evidence of prosociality in Study 2 where they had to select between two simultaneously presented apparatuses (similar to PC tasks).

Also in Study 1, greater experience with the task led Actors to be less willing to pull the handle and operate the apparatus for the 0/0 and 0/0(1) payoff distributions, but not for 0/1. Actors thus learned to reduce their pulling when neither they nor a Recipient obtained a reward, but not when a payoff could be obtained from the Recipient bin (likely by a Recipient). This would predict that greater experience with the *0/1 v. 0/0* condition from Study 2 should lead Actors to reduce their choices of 0/0 but not 0/1. The likelihood of selecting the prosocial outcome in the *0/1 v. 0/0* condition should have increased as the study progressed, but this did not happen. If anything, with greater experience Actors simply became more likely to do nothing.

Subject animals in Study 1 showed in a number of ways that they understood how to use the apparatus so as to enable access to food in the Recipient bin, and the causal affordances of the apparatuses in Study 2 were identical to those of the apparatus used in Study 1. Thus, it is unlikely that the reduction in prosociality in Study 2 was due to an inability to understand that pulling the handle for this kind of apparatus would make food available to the Recipient, and it was more likely due to the introduction of an additional apparatus (i.e., a second payoff choice). There are many reasons why this could have influenced Actors' behavior. The addition of a second apparatus may have distracted the animals in Study 2 and prevented them from attending to the Recipient's payoff. Alternatively, evaluating the merits of one payoff outcome (choice) in Study 1 may be much more straightforward and less noisy a process than evaluating two choices and comparing them simultaneously, as animals may have had to do in Study 2. Regardless, these results imply that a particular kind of task complexity (i.e., number of discrete choices) may be particularly problematic for laboratory studies of prosociality in captive animals. Our results suggest that the difference in the results obtained across PC and IH tasks isn't due to differences in the causal complexity of the individual components of the experimental tasks (e.g., the causal affordances of the apparatuses). Instead, differences in results may be due rather to differences in the number of alternative choices the two kinds of tasks present to subject animals.

Note that we do not use task complexity to mean task difficulty, and we do not know whether differences in prosocial behavior across tasks of different complexity is due to differences in how cognitively demanding subject animals found these tasks to be. It is possible that more complex tasks are indeed more cognitively demanding than are simpler tasks, perhaps because they require attention to and tracking of more objects and locations, but it is also possible that more complex tasks are simple less ecologically relevant or less interesting to animals. Furthermore, while our results are consistent with the hypothesis that more-complex tasks are more cognitively demanding than are less-complex tasks (in addition to other hypotheses described above), they do not suggest that all tasks involving two choices will fail to elicit prosocial behavior in captive chimpanzees. Instead, our results predict that within any given kind of apparatus or task paradigm, tasks with relatively more choices or components might be less effective at eliciting prosocial behavior.

These results are interesting to consider in light of recent findings showing that chimpanzees displayed a systematic bias towards selecting prosocial outcomes in a variant of the PC task in which Actors obtain food when delivering food to Recipients [14], a result that appears to contradict those of the *1/1 v. 1/0* condition that we used in Study 2 and also the results of prior studies using the PC task [10]–[13], [37]. In this study, subject chimpanzees were provided with a bucket containing two kinds of tokens, and subjects could transfer one kind of token to the experimenter to confer a 1/1 outcome (i.e., to provide food to a Recipient) and the other kind of token to confer a 1/0 outcome. It is possible that the structure of this task allows subjects to consider tokens serially rather than simultaneously, and thus allowed animals to evaluate individual tokens (i.e., individual payoff outcomes/choices) in isolation, similar to how animals in Study 1 evaluated individual payoff outcomes in isolation. It is possible that if two tokens were presented in parallel to animals by an experimenter in a more binary-choice situation (as in Study 2), animals would show a reduced tendency to select the 1/1 token. This could potentially account for other findings where chimpanzees display prosocial behavior in situations where they can evaluate one possible choice at a time out of a set of provided choices, such as a result where Yamamoto and colleagues provided subject chimpanzees with a set of possible tools and observed that subjects tended to pass the correct tool to a Recipient who needed it to access a food reward [20]. This would be a good test of the hypothesis that the number of discrete simultaneous choices with which animals are presented influences the likelihood of observing prosocial behavior.

It is also possible that the use of tokens themselves might also be crucial for understanding differences in observations of prosocial behavior across published studies, and differences in the use of this methodology could underlie controversial inconsistencies in how chimpanzees' behave in recent studies of the Ultimatum Game [54], [55]. Future research should explicitly test whether the use of tokens impacts prosocial behavior, in the same way that we have here tested other methodological hypotheses. One final possibility is that differences between captive populations tested may play a role [56]. There is evidence for variation in cooperative behavior across populations of wild chimpanzees, with, for example, hunting by chimpanzees in the Taï forest being collaborative and marked by exchange of meat for mating opportunities [2], [50], while hunting in Kibale National Park was less consistent with collaboration or meat-for-mating exchanges [57]. However, differences in cooperation across captive populations is not well understood.

Our results are also inconsistent with studies finding inequity aversion in captive chimpanzees [23]. If Actors in the current study had been averse to disadvantageous inequity, we would have expected them to pull the handle less for 1/3 trials than for 1/1 trials, a pattern similar to one reported when capuchin monkeys (*Cebus apella*) were presented with a forced-choice between 1/1 and 1/3 [58]. However, our chimpanzee Actors appeared insensitive to the difference between these payoff distributions, even though animals showed that they understood the apparatus used in Study 1. It has been argued that it is necessary for subject animals to be engaged in an experimental task for a response to inequity to be elicited [23], and consistent with this Actors in our studies are engaged in a task. However, the structure of our task is distinctly different from the standard task that elicits inequity aversion, in that our task involves no token exchange and the subject animal directly creates the inequitable outcome by delivering the rewards to themselves and a conspecific [23] (rather than indirectly creating the inequity through an experimenter, as in many IA studies). It is possible that the use of tokens and the role of the experimenter is required for the phenomenon to hold, but it remains a tantalizing theoretical question why laboratory elicitation of inequity aversion in chimpanzees might be constrained to these particular task designs [28], [29].

These results suggest that a task using a single apparatus may be better at eliciting patterns of prosocial behavior in captive chimpanzees than would be a task using two copies of the same apparatus, and in this way the present studies suggest that asymmetries in evidence for prosocial behavior across different experimental tasks may be due to asymmetries in task complexity (defined as the number of components or choices included in a task design). Precisely why more complex tasks may inhibit prosocial behavior in these animals cannot be determined from the present study, and many other differences in methodology might also influence chimpanzee prosociality in laboratory tasks (e.g., the value or kind of food rewards, the use of tokens). Future work should explore these issues. However, a larger matter still looms: despite the plausible need for cooperation in both captive and wild chimpanzee social groups, evidence for chimpanzee prosociality in the laboratory is still weak relative to evidence from the wild. A primary benefit of laboratory studies is that they permit the investigation of mechanisms and motivations underlying chimpanzees' naturally-occurring prosocial behavior, but to do this requires experimental tasks that capture prosocial behavior in such a way that costs and benefits can be manipulated in a controlled manner. The methods used here describe one way to approach this problem, while also employing a task that more closely emulates the freedom for partner choice that exists in the wild. Making experiments progressively more naturalistic should be a goal of laboratory work, and it will allow more powerful generalization of laboratory results to the behavior of wild chimpanzees.

## Methods

This study protocol was approved by the Animal Research Committee of the University of California, Los Angeles (ARC approval permit #2011-036-01). Animals were drawn from a captive population of 175 chimpanzees housed at the KCCMR in Bastrop, Texas. Animals were not food-deprived, and had ad libitum access to food and water throughout the study. Supplementary fruits and vegetables were provided to animals once daily. Animals were housed in social group enclosures with both exterior and interior areas, and all animals participated in a behavioral management program to ensure their mental health and well-being. This program includes daily environmental enrichment procedures in which animals are provisioned in both indoor and outdoor areas with enrichment devices for resting and climbing (e.g., grass ground cover, multi-level wooden platforms, hammocks, hanging tires, brachiation bars, utility poles, ropes and cargo nets, barrels, and large culverts), along with additional foraging apparatuses (e.g., kong toys filled with food) which were changed regularly. The study did not interfere with animals' normal activities except when they were isolated during the Knowledge Probe in Study 1. The Knowledge Probe was only begun when animals did not show stress at being isolated, it lasted about 30 minutes, and it was halted if animals began to show any signs of distress. Animals were isolated in the exterior areas of their home enclosure, and had unconstrained access to light, food, water, and enrichment. No animals were harmed.

### Study 1

#### Participants

Many participants had previously taken part in cognitive experiments, and some in prior experiments on prosocial behavior [10], [37]. Animals were housed in one all-female or one all-male social group of 6 or 7 animals whose membership had been stable for several years, and had access to food and water throughout the day. Groups were entirely composed of adults.

#### Apparatus

Actor animals were able to deliver payoffs to themselves and/or other group members by using an apparatus consisting of two plastic food bins that were anchored to the enclosure. When a handle was pulled by the Actor both bins pivoted upwards, allowing animals inside the enclosure to obtain food placed in the bins, but the distance between the bins prevented one animal from pulling the handle and obtaining rewards from both bins (see Figure 1). Only one handle was provided, and when the handle was released both bins returned to their original position and rewards were not accessible, ensuring that the Actor could obtain rewards from only the Actor bin (and not the Recipient bin).

#### Task comprehension

During pilot testing animals were willing to pull the handle and able to retrieve rewards from the Actor bin (i.e., animals were not scared of the apparatus), so instead of using a formal training procedure we evaluated whether animals' behavior changed as a function of experience (i.e. trial number). These results indicate that Actors understood that the food in both the Actor bin and the Recipient bin was obtainable, and that they conditioned their behavior on the presence of this food (see Study 1 Results). To further assay animals' understanding of the task, at the conclusion of Study 1 we also conducted a Knowledge Probe where we explored whether Actors were more likely to pull the handle when the apparatus was modified to allow them to directly access food from the Recipient bin (see below).

#### Testing procedure

Testing consisted of sequential blocks of six randomized trials, with each trial corresponding to a different payoff distribution. This counterbalanced the payoff distributions by presenting each distribution once every 6 trials (on average). Each trial type appeared no more than twice in succession, and did not predict the next trial type. The number of blocks presented each day varied based on each group's availability for testing. Trials were run whenever a potential Actor was present, and animals were coaxed to participate by verbal calls and displaying the available food rewards.

A trial did not start unless (1) 30 seconds had passed from the start of the previous trial, (2) a potential Actor was near the testing area, (3) the apparatus was at rest in the No Access position (see Figure 1), and (4) any payoffs remaining from the previous trial had been removed. A trial lasted no less than 30 seconds, and ended if the animal did not pull within that period or pulled before all payoffs were placed. The experimenter called the Actor's attention and waited until Actors looked in his direction as he held up the payoff and then placed it by hand into the appropriate bin (if the trial called for zero payoffs the experimenter only touched the bin).

#### Knowledge Probe

At the conclusion of Study 1 we modified the apparatus with a longer handle to allow Actors to pull the handle and climb down to obtain food from the Recipient bin. We then isolated animals and presented them with trials using the 0/1, 1/1, and 0/0 payoff distributions using this modified apparatus. If Actors understood that pulling the handle caused food in the Recipient bin to become accessible, they should be more likely to pull the handle in the Knowledge Probe than in the Test trials for the 0/1 payoff distribution, but not for the 1/1 and 0/0 payoff distributions. We conducted the Knowledge Probe at the end of Study 1 to avoid training animals that they could directly obtain food for themselves by pulling the handle during 0/1 trials, which could appear as prosocial behavior if the Testing trials followed the Knowledge Probe. Four of the subject animals (two male, two female) were willing to be isolated for the Knowledge Probe.

### Study 2

#### Participants

One of the participating social groups included animals ranging from one juvenile to adults, while the other two social groups included mostly older adults. All groups were mixed-sex. The composition of two groups from which participants were drawn had been stable for several years, while the third group was newly formed (this group contributed only one participant). These groups were entirely different from the groups participating in Study 1.

#### Procedure

The procedure was identical to that of Study 1, but a familiarization procedure was employed because the apparatus was larger and had more parts, and we wanted to ensure that animals were not afraid of it. Prior to testing, subjects received 40 counterbalanced trials from the *1/1 v. 0/1* condition. Testing consisted of blocks of eights trials, each containing four sets of two trials. Each of the fours sets of two trials used one of the four basic payoff distributions, but counterbalanced the side of presentation of specific payoff outcomes (e.g. one trial would load 1/0 on the left and 1/1 on the right, and the second would load 1/1 on the left and 1/0 on the right). Within each block, the order of these 8 trials was randomized. No trial appeared more than twice in succession, and no trial type predicted the subsequent trial type.

At the start of a trial the experimenter drew the Actor's attention verbally, and waited until the animal looked in his direction as he held up each reward and loaded it by hand first into the Recipient bins, followed by the Actor bins (see Figure 4). The Test Phase began when the experimenter inserted the handles into the enclosure, making them accessible to the Actor (see Figure 4).

## Supporting Information

Figure S1 **Study 1, Knowledge Probe.** Plot of regression models (Table S4) of the effect of *Actor's Trial Number* on actors' willingness to pull the handle. Each plot models the behavior of one individual. Solid lines reflect the estimated probability that an individual will pull the handle when presented with a 0/1 payoff distribution, as a function of how many 0/1 trials they have received. Dashed lines reflect the estimated probability that an individual will pull the handle when presented with a 0/0 payoff distribution, as a function of how many 0/0 trials they have received. Shaded areas reflect 95% confidence intervals. See Table S6 and Table S7 for model coefficients and variance estimates. For subjects 201, 303, and 302 the estimates suggest that animals were more likely to pull the handle on the first 0/1 trial (solid line) than on the first 0/0 trial (dashed line). This suggests that the animals were able to figure out from the beginning of the Knowledge Probe that they would now be able to obtain food on 0/1 trials. For Subject 202, the estimates are somewhat counterintuitive for the first few trials, with a rather high likelihood of pulling the handle on 0/0 trials but a low likelihood on 0/1 trials, but by trial #6 the pattern displayed by the other subjects emerges and remains consistent. This suggests that Subject 202 was somehow confused at the start of the Knowledge Probe, and it took a few trials to figure out that food was now accessible on 0/1 trials but not on 0/0 trials. However, the overall pattern is one of animals quite quickly adapting their behavior to fit the modified apparatus in the Knowledge Probe, and maximizing their food payoffs by pulling the handle on 0/1 trials.
(TIF)

Figure S2 **Study 1, Knowledge Probe.** Analysis of 0/0 trials. Animals were presented with three different payoff distributions in the Knowledge Probe: 0/1, 1/1, and 0/0. If Actor animals understood that pulling the handle made food in the Recipient bin accessible, they should be more willing to pull the handle in the Knowledge Probe than the Test trials when food is placed only in the Recipient bin (i.e., the 0/1 payoff distribution). This is because pulling the handle allows them to obtain food for themselves in the Knowledge Probe, but not in the Test trials. However, for the 1/1 payoff distribution animals should be near-ceiling in their willingness to pull the handle both in the Test and Knowledge Probe, because in both sets of trials they obtain food for themselves. Similarly, in the 0/0 payoff distribution animals should be near-floor in their willingness to pull the handle in both the Test and Knowledge Probe, because in both sets of trials they cannot obtain food for themselves. Results showed that these four subject animals were indeed more likely to pull the handle for the 0/1 payoff distribution in the Knowledge Probe than in the Test trials (see Figure 3 in main text). Additionally, animals pulled the handle for the 1/1 payoff distribution on every trial in both the Knowledge Probe and Test trials. Actor animals also pulled the handle at very low rates for the 0/0 payoff distribution in both the Knowledge Probe and Test trials, and at comparable rates across both sets of trials (see accompanying Figure S2). It is true that subject 202 did pull the handle more frequently on 0/0 Knowledge trials than 0/0 Test trials, but moving from the Test trials to the Knowledge Probe has a much greater impact on subject 202's rates of pulling the handle for the 0/1 payoff distribution (main text Figure 2) than the 0/0 payoff distribution (Figure S2).
(TIF)

Table S1 **Study 1, Total number of observations contributed by each subject in Study 1, along with demographic data on subjects.** Total N/Average N per animal, for Low dominance: 92/30.7. Total N/Average N per animal, for Medium dominance: 542/136.8. Total N/Average N per animal, for High dominance: 122/40.7.
(DOCX)

Table S2 **Study 1, Multi-level logistic regression models replicating the results displayed in** **Figure 2A** **, controlling for subject identity (i.e., non-independence in the data).** Animals pulled the handle on every 1/3 trial.
(DOCX)

Table S3 **Study 1, Robustness analysis.** Table provides the coefficients for the parameter *0/1* in Table S3 above (which models how animals' behavior changes when switching from a 0/0 trial to a 0/1 trial), when dropping out each of the animals in the sample one at a time. One of the animals contributed no data to this condition (206), but for most of the other animals excluding their data does not change the valence of the coefficient, nor does it dramatically change the magnitude of the coefficient (which remains larger than it's standard error). This means that even though there is substantial variation in the amount of data each animal contributes to the sample, the pattern is not likely to be entirely driven by any one animal.
(DOCX)

Table S4 **Study 1, Models of the effect of** ***Actor's Trial Number*** **on actors' willingness to pull the handle and operate the apparatus in Study 1, represented in** **Figure 2B** **.**
(DOCX)

Table S5 **Study 1, Knowledge Probe.** Multilevel logistic regression for Knowledge Probe. Effect of moving from the Test trials into the Knowledge Probe on Actor's frequency of pulling the handle. The models below ask how the binary variable *Trial was in Knowledge Probe* predicts the probability that animals pulled the handle. For the 0/0 payoff, animals were not more likely to pull the handle if the trial was in the Knowledge Probe, as the coefficient for the parameter is smaller than its standard error. However, for the 0/1 payoff, animals were much more likely to pull the handle if the trial was in the Knowledge Probe, as the coefficient for the parameter is much larger than its standard error. This corresponds to an Odds Ratio of 44.6, meaning that Actors were 44 times more likely to pull the handle in the Knowledge Probe than in the Test trials. The coefficient for the Random Effect parameter is also somewhat larger than its standard error, suggesting that there may be some variation in this pattern across individuals.
(DOCX)

Table S6 **Study 1, Knowledge Probe.** Regression model for Figure S1. This model predicts the Actor's probability of pulling the handle as a function of three predictors: *Payoff_is_0/1*, codes whether the payoff on a given trial was 0/1 or 0/0 (with 0/1 coded as ‘2’ and 0/0 coded as ‘1’). *Payoff_trialnum*, codes the trial number of each trail within a particular payoff distribution (i.e., whether this is the first 0/1 trial, the second, etc.). *Payoff_trialnum X Payoff_is_0/1*, an interaction term for the other two parameters. This is the most interpretable and interesting term in the model, and indicates how the difference in pulling across the 0/1 and 0/0 payoffs changes as a function of trial number (i.e., experience in the task). The model also allows these parameters to vary across the four individuals tested in the Knowledge Probe. However, with only four individuals the model has difficulty estimating the variance of these predictors across clusters (individuals), and the raw coefficients and standard deviations are difficult to interpret. In particular, the interaction term is positive but has a large variance. Table S7 provides a similar model that does not allow these parameters to vary across individuals, making the raw estimates for coefficients and variance more interpretable. These regressions were performed in the R statistical computing environment using Stan, a Hamiltonian Monte Carlo sampler, and glmer2stan a convenience package for generating generalized linear mixed model code for Stan.
(DOCX)

Table S7 **Study 1, Knowledge Probe.** Regression model for Figure S1, excluding varying estimates for each Actor. Similar model as in Table S6, but without allowing the parameters to vary across individuals. The interaction term is the most informative here, and the coefficient for this predictor is greater than zero. This was not obvious from the prior model in Table S5, because the parameter was allowed to vary across a small number of individuals (only four).
(DOCX)

Table S8 **Study 2, Total number of observations contributed by each subject in Study 2, along with demographic data on subjects.** Total N/Average N per animal, for Low dominance: 680/136. Total N/Average N per animal, for Medium dominance: 244/244. Total N/Average N per animal, for High dominance: 281/140.5.
(DOCX)

Table S9 **Study 2, Regression models of the effect of** ***Actor's Trial Number*** **on actors' likelihood of choosing the prosocial option in Study 2, represented in** **Figure 5B** **.**
(DOCX)

Table S10 **Study 2, Regression models of the effect of** ***Actor's Trial Number*** **on actors' likelihood of choosing neither handle in the 0/1 v. 0/0 condition of Study 2, represented in** **Figure 5B** **.**
(DOCX)
