# Supplementary figures and images for: Task Design Influences Prosociality in Captive Chimpanzees (Pan troglodytes)
Source: PLoS One. 2014 Sep 5;9(9):e103422. doi: 10.1371/journal.pone.0103422 (PMC4156467; doi:10.1371/journal.pone.0103422)

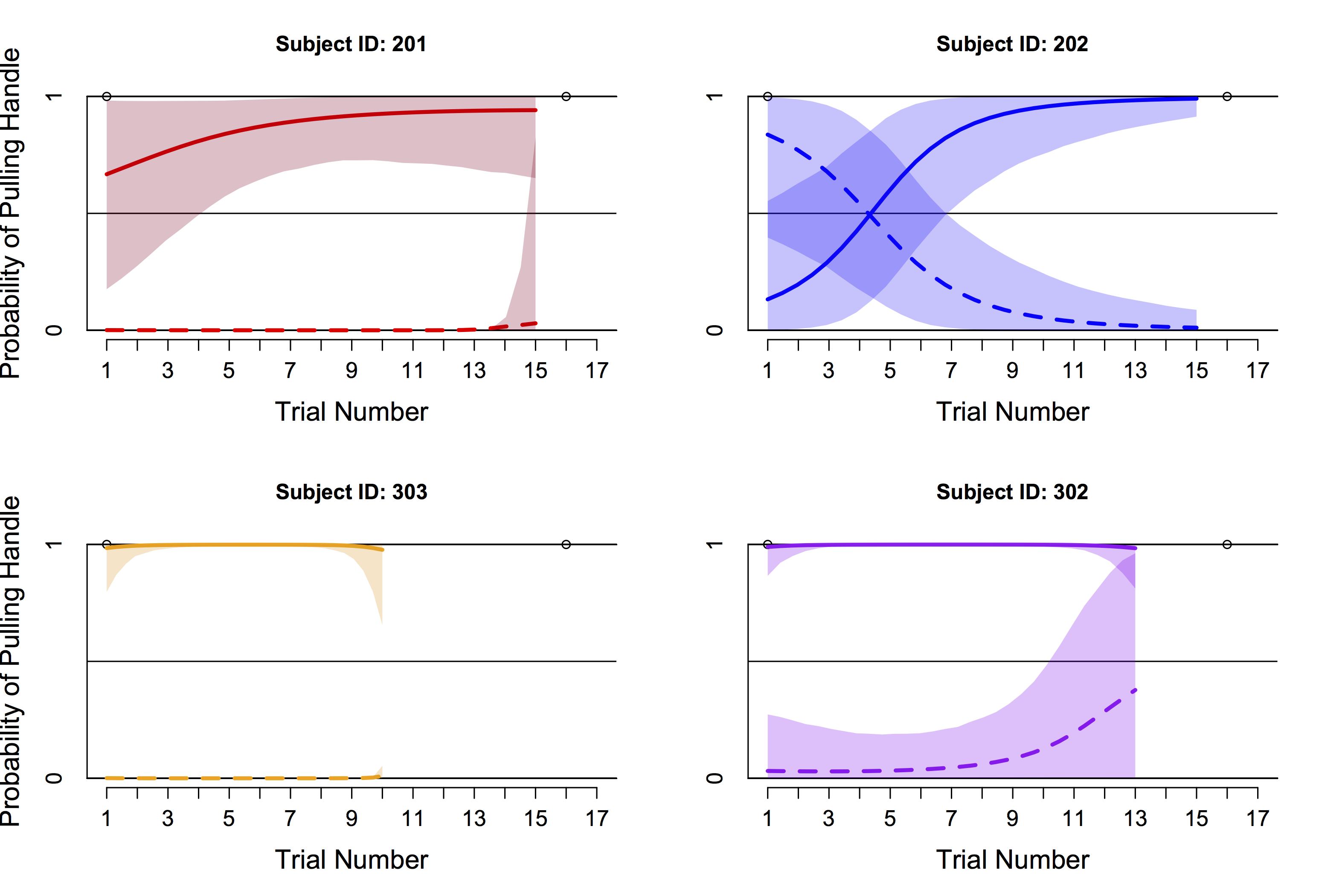

Supplement: Figure S1 — Study 1, Knowledge Probe. Plot of regression models (Table S4) of the effect of Actor's Trial Number on actors' willingness to pull the handle. Each plot models the behavior of one individual. Solid lines reflect the estimated probability that an individual will pull the handle when presented with a 0/1 payoff distribution, as a function of how many 0/1 trials they have received. Dashed lines reflect the estimated probability that an individual will pull the handle when presented with a 0/0 payoff distribution, as a function of how many 0/0 trials they have received. Shaded areas reflect 95% confidence intervals. See Table S6 and Table S7 for model coefficients and variance estimates. For subjects 201, 303, and 302 the estimates suggest that animals were more likely to pull the handle on the first 0/1 trial (solid line) than on the first 0/0 trial (dashed line). This suggests that the animals were able to figure out from the beginning of the Knowledge Probe that they would now be able to obtain food on 0/1 trials. For Subject 202, the estimates are somewhat counterintuitive for the first few trials, with a rather high likelihood of pulling the handle on 0/0 trials but a low likelihood on 0/1 trials, but by trial #6 the pattern displayed by the other subjects emerges and remains consistent. This suggests that Subject 202 was somehow confused at the start of the Knowledge Probe, and it took a few trials to figure out that food was now accessible on 0/1 trials but not on 0/0 trials. However, the overall pattern is one of animals quite quickly adapting their behavior to fit the modified apparatus in the Knowledge Probe, and maximizing their food payoffs by pulling the handle on 0/1 trials. (TIF) [file pone.0103422.s001.tif]

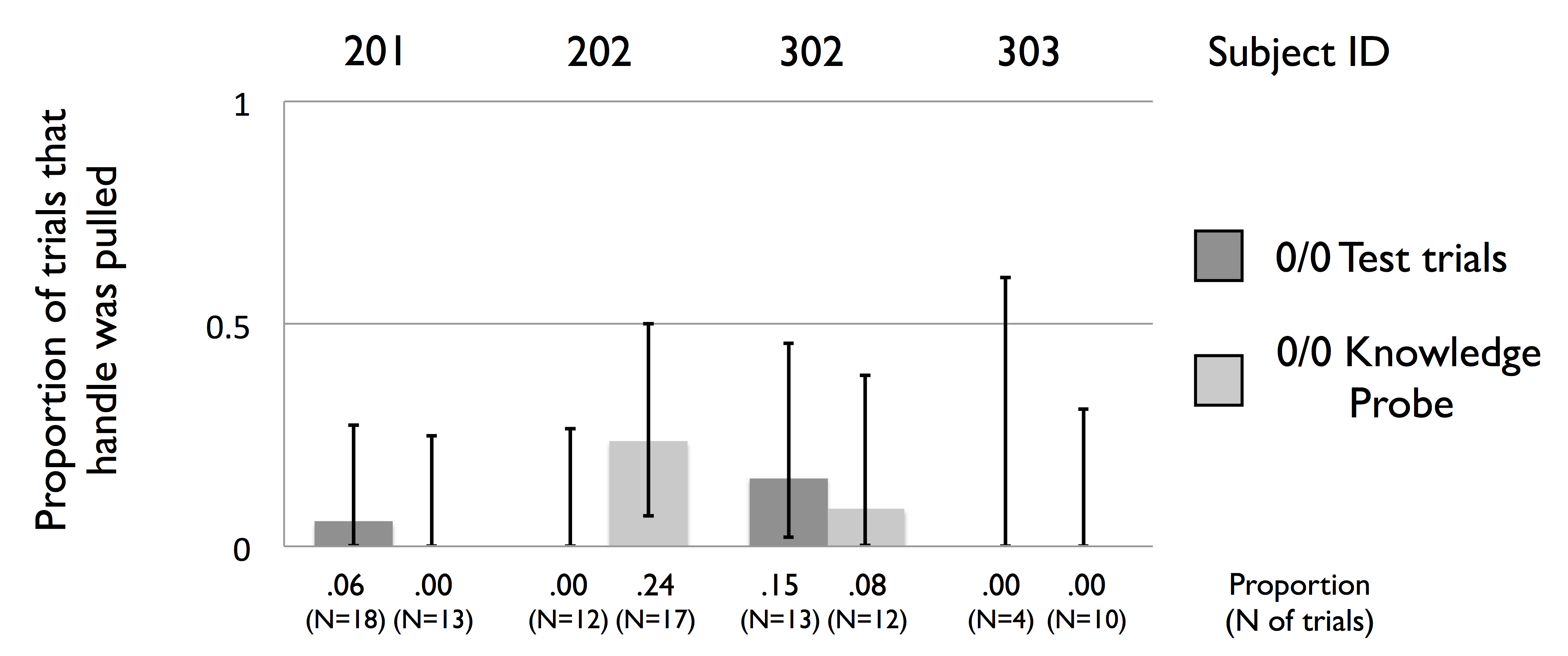

Supplement: Figure S2 — Study 1, Knowledge Probe. Analysis of 0/0 trials. Animals were presented with three different payoff distributions in the Knowledge Probe: 0/1, 1/1, and 0/0. If Actor animals understood that pulling the handle made food in the Recipient bin accessible, they should be more willing to pull the handle in the Knowledge Probe than the Test trials when food is placed only in the Recipient bin (i.e., the 0/1 payoff distribution). This is because pulling the handle allows them to obtain food for themselves in the Knowledge Probe, but not in the Test trials. However, for the 1/1 payoff distribution animals should be near-ceiling in their willingness to pull the handle both in the Test and Knowledge Probe, because in both sets of trials they obtain food for themselves. Similarly, in the 0/0 payoff distribution animals should be near-floor in their willingness to pull the handle in both the Test and Knowledge Probe, because in both sets of trials they cannot obtain food for themselves. Results showed that these four subject animals were indeed more likely to pull the handle for the 0/1 payoff distribution in the Knowledge Probe than in the Test trials (see Figure 3 in main text). Additionally, animals pulled the handle for the 1/1 payoff distribution on every trial in both the Knowledge Probe and Test trials. Actor animals also pulled the handle at very low rates for the 0/0 payoff distribution in both the Knowledge Probe and Test trials, and at comparable rates across both sets of trials (see accompanying Figure S2). It is true that subject 202 did pull the handle more frequently on 0/0 Knowledge trials than 0/0 Test trials, but moving from the Test trials to the Knowledge Probe has a much greater impact on subject 202's rates of pulling the handle for the 0/1 payoff distribution (main text Figure 2) than the 0/0 payoff distribution (Figure S2). (TIF) [file pone.0103422.s002.tif]
