# Supplementary material for: Task Design Influences Prosociality in Captive Chimpanzees (Pan troglodytes)
Source: PLoS One. 2014 Sep 5;9(9):e103422. doi: 10.1371/journal.pone.0103422 (PMC4156467; doi:10.1371/journal.pone.0103422)
Supplement: Table S1 — Study 1, Total number of observations contributed by each subject in Study 1, along with demographic data on subjects. Total N/Average N per animal, for Low dominance: 92/30.7. Total N/Average N per animal, for Medium dominance: 542/136.8. Total N/Average N per animal, for High dominance: 122/40.7. (DOCX) [file pone.0103422.s003.docx]

**Table S1:** Total number of observations contributed by each subject in Study 1, along with demographic data on subjects.

Total N / Average N per animal, for Low dominance: 92 / 30.7

Total N / Average N per animal, for Medium dominance: 542 / 136.8

Total N / Average N per animal, for High dominance: 122 / 40.7

| **Enclosure** | **Subject** | **Total *n* observations for this actor** | **Sex** | **Age (in years)** | **Birthplace,**  **Rearing history** | **Dominance level in group** |
| --- | --- | --- | --- | --- | --- | --- |
| 1 | 201 | 99 | F | 28.41 | Captive,  nursery raised | Medium |
| 1 | 202 | 59 | F | 29.01 | Captive, mother raised | Low |
| 1 | 203 | 32 | F | 32.34 | Captive,  nursery raised | Low |
| 1 | 204 | 13 | F | 49.46 | Wild,  unknown | Medium |
| 1 | 205 | 3 | F | 43.36 | Wild,  unknown | High |
| 1 | 206 | 1 | F | 40.46 | Wild,  unknown | Low |
| 2 | 301 | 124 | M | 20.40 | Captive, mother raised | Medium |
| 2 | 302 | 89 | M | 18.55 | Captive, mother raised | High |
| 2 | 303 | 30 | M | 21.07 | Captive, mother raised | High |
| 2 | 306 | 5 | M | 22.01 | Captive, mother raised | Medium |
