# Supplementary material for: Task Design Influences Prosociality in Captive Chimpanzees (Pan troglodytes)
Source: PLoS One. 2014 Sep 5;9(9):e103422. doi: 10.1371/journal.pone.0103422 (PMC4156467; doi:10.1371/journal.pone.0103422)
Supplement: Table S2 — Study 1, Multi-level logistic regression models replicating the results displayed in Figure 2A , controlling for subject identity (i.e., non-independence in the data). Animals pulled the handle on every 1/3 trial. (DOCX) [file pone.0103422.s004.docx]

**Table S2:** Multi-level logistic regression models replicating the results displayed in Figure 2A, controlling for subject identity (i.e., non-independence in the data). Animals pulled the handle on every 1/3 trial.

| DV: Actor Pulled Handle | Coef. (SE) |
| --- | --- |
| 0 / 0 (1) | .34 (.67) |
| 0 / 1 | 1.22 (.61) |
| 1 / 0 | 5.96 (.77) |
| 1 / 1 | 7.42 (1.16) |
| 1 / 3 | 24.1 (n/a) |
| Random effect | .30 (.41) |
| Constant | -2.06 |
