# Supplementary material for: Task Design Influences Prosociality in Captive Chimpanzees (Pan troglodytes)
Source: PLoS One. 2014 Sep 5;9(9):e103422. doi: 10.1371/journal.pone.0103422 (PMC4156467; doi:10.1371/journal.pone.0103422)
Supplement: Table S3 — Study 1, Robustness analysis. Table provides the coefficients for the parameter 0/1 in Table S3 above (which models how animals' behavior changes when switching from a 0/0 trial to a 0/1 trial), when dropping out each of the animals in the sample one at a time. One of the animals contributed no data to this condition (206), but for most of the other animals excluding their data does not change the valence of the coefficient, nor does it dramatically change the magnitude of the coefficient (which remains larger than it's standard error). This means that even though there is substantial variation in the amount of data each animal contributes to the sample, the pattern is not likely to be entirely driven by any one animal. (DOCX) [file pone.0103422.s005.docx]

**Table S3:** Robustness analysis. Table provides the coefficients for the parameter *0/1* in Table S3 above (which models how animals’ behavior changes when switching from a 0/0 trial to a 0/1 trial), when dropping out each of the animals in the sample one at a time. One of the animals contributed no data to this condition (206), but for most of the other animals excluding their data does not change the valence of the coefficient, nor does it dramatically change the magnitude of the coefficient (which remains larger than it’s standard error). This means that even though there is substantial variation in the amount of data each animal contributes to the sample, the pattern is not likely to be entirely driven by any one animal.

| Actor animal excluded from analysis | *N* observations for this Actor | *N* observations remaining in sample | Coefficient for moving from  0/0 to 0/1 | Standard error |
| --- | --- | --- | --- | --- |
| none |  | 150 | 1.24 | .62 |
| 201 | 35 | 115 | .87 | .75 |
| 202 | 22 | 128 | 1.22 | .62 |
| 203 | 10 | 140 | 1.22 | .62 |
| 204 | 4 | 146 | 1.42 | .69 |
| 205 | 1 | 149 | 1.26 | .62 |
| 206 | 0 |  |  |  |
| 301 | 43 | 107 | 1.03 | .65 |
| 302 | 26 | 124 | 1.98 | .83 |
| 303 | 7 | 143 | 1.12 | .63 |
| 306 | 2 | 148 | 1.12 | .63 |
| 201 & 302 | 61 | 89 | 2.08 | 1.24 |
