# Supplementary material for: Task Design Influences Prosociality in Captive Chimpanzees (Pan troglodytes)
Source: PLoS One. 2014 Sep 5;9(9):e103422. doi: 10.1371/journal.pone.0103422 (PMC4156467; doi:10.1371/journal.pone.0103422)
Supplement: Table S4 — Study 1, Models of the effect of Actor's Trial Number on actors' willingness to pull the handle and operate the apparatus in Study 1, represented in Figure 2B . (DOCX) [file pone.0103422.s006.docx]

**Table S4:** Models of the effect of *Actor’s Trial Number* on actors’ willingness to pull the handle and operate the apparatus in Study 1, represented in Figure 2B.

| DV: Actor Pulled Handle | Model 1 | Model 2 | Model 3 | Model 4 | Model 5 |
| --- | --- | --- | --- | --- | --- |
|  | 0 / 0 | 0/0(1) | 0 / 1 | 1 / 0 | 1 / 1 |
|  | Coef. (SE) | Coef. (SE) | Coef. (SE) | Coef. (SE) | Coef. (SE) |
| Actor’s Trial Number | -.079 (.047) | -.092 (.042) | .002 (.01) | .002 (.01) | .02 (.03) |
| Random Effect | 1.01 (1.24) | .001 (.61) | .51 (.69) | .001 (.02) | .003 (.11) |
| Constant | -.91 | -.44 | -1.89 | 2.80 | 3.64 |
