# Supplementary material for: Task Design Influences Prosociality in Captive Chimpanzees (Pan troglodytes)
Source: PLoS One. 2014 Sep 5;9(9):e103422. doi: 10.1371/journal.pone.0103422 (PMC4156467; doi:10.1371/journal.pone.0103422)
Supplement: Table S5 — Study 1, Knowledge Probe. Multilevel logistic regression for Knowledge Probe. Effect of moving from the Test trials into the Knowledge Probe on Actor's frequency of pulling the handle. The models below ask how the binary variable Trial was in Knowledge Probe predicts the probability that animals pulled the handle. For the 0/0 payoff, animals were not more likely to pull the handle if the trial was in the Knowledge Probe, as the coefficient for the parameter is smaller than its standard error. However, for the 0/1 payoff, animals were much more likely to pull the handle if the trial was in the Knowledge Probe, as the coefficient for the parameter is much larger than its standard error. This corresponds to an Odds Ratio of 44.6, meaning that Actors were 44 times more likely to pull the handle in the Knowledge Probe than in the Test trials. The coefficient for the Random Effect parameter is also somewhat larger than its standard error, suggesting that there may be some variation in this pattern across individuals. (DOCX) [file pone.0103422.s007.docx]

**Table S5:** Multilevel logistic regression for Knowledge Probe. Effect of moving from the Test trials into the Knowledge Probe on Actor’s frequency of pulling the handle. The models below ask how the binary variable *Trial was in Knowledge Probe* predicts the probability that animals pulled the handle. For the 0/0 payoff, animals were not more likely to pull the handle if the trial was in the Knowledge Probe, as the coefficient for the parameter is smaller than its standard error.

However, for the 0/1 payoff, animals were much more likely to pull the handle if the trial was in the Knowledge Probe, as the coefficient for the parameter is much larger than its standard error. This corresponds to an Odds Ratio of 44.6, meaning that Actors were 44 times more likely to pull the handle in the Knowledge Probe than in the Test trials. The coefficient for the Random Effect parameter is also somewhat larger than its standard error, suggesting that there may be some variation in this pattern across individuals.

| DV: pulled handle | 0 / 1 payoff  N (subjects) = 4  N (observations) = 85 | 0 / 0 payoff  N (subjects) = 4  N (observations) = 92 |
| --- | --- | --- |
|  | Coef. (SE) | Coef. (SE) |
| Trial was in Knowledge Probe (0=No, 1=Yes) | 3.80 (.74) | .61 (.76) |
| Constant | -1.77 (.62) | -2.71 (.65) |
| Random Effect | .82 (.54) | .23 (1.02) |
