# Supplementary material for: Task Design Influences Prosociality in Captive Chimpanzees (Pan troglodytes)
Source: PLoS One. 2014 Sep 5;9(9):e103422. doi: 10.1371/journal.pone.0103422 (PMC4156467; doi:10.1371/journal.pone.0103422)
Supplement: Table S6 — Study 1, Knowledge Probe. Regression model for Figure S1. This model predicts the Actor's probability of pulling the handle as a function of three predictors: Payoff_is_0/1, codes whether the payoff on a given trial was 0/1 or 0/0 (with 0/1 coded as ‘2’ and 0/0 coded as ‘1’). Payoff_trialnum, codes the trial number of each trail within a particular payoff distribution (i.e., whether this is the first 0/1 trial, the second, etc.). Payoff_trialnum X Payoff_is_0/1, an interaction term for the other two parameters. This is the most interpretable and interesting term in the model, and indicates how the difference in pulling across the 0/1 and 0/0 payoffs changes as a function of trial number (i.e., experience in the task). The model also allows these parameters to vary across the four individuals tested in the Knowledge Probe. However, with only four individuals the model has difficulty estimating the variance of these predictors across clusters (individuals), and the raw coefficients and standard deviations are difficult to interpret. In particular, the interaction term is positive but has a large variance. Table S7 provides a similar model that does not allow these parameters to vary across individuals, making the raw estimates for coefficients and variance more interpretable. These regressions were performed in the R statistical computing environment using Stan, a Hamiltonian Monte Carlo sampler, and glmer2stan a convenience package for generating generalized linear mixed model code for Stan. (DOCX) [file pone.0103422.s008.docx]

**Table S6:** Regression Model for Figure S1.

This model predicts the Actor’s probability of pulling the handle as a function of three predictors:

*Payoff_is_0/1*, codes whether the payoff on a given trial was 0/1 or 0/0 (with 0/1 coded as ‘2’ and 0/0 coded as ‘1’).

*Payoff_trialnum,* codes the trial number of each trail within a particular payoff distribution (i.e., whether this is the first 0/1 trial, the second, etc.).

*Payoff_trialnum X Payoff_is_0/1*, an interaction term for the other two parameters. This is the most interpretable and interesting term in the model, and indicates how the difference in pulling across the 0/1 and 0/0 payoffs changes as a function of trial number (i.e., experience in the task).

The model also allows these parameters to vary across the four individuals tested in the Knowledge Probe. However, with only four individuals the model has difficulty estimating the variance of these predictors across clusters (individuals), and the raw coefficients and standard deviations are difficult to interpret. In particular, the interaction term is positive but has a large variance. Table S7 provides a similar model that does not allow these parameters to vary across individuals, making the raw estimates for coefficients and variance more interpretable.

These regressions were performed in the R statistical computing environment using Stan, a Hamiltonian Monte Carlo sampler, and glmer2stan a convenience package for generating generalized linear mixed model code for Stan.

| **Level 1 Estimates** | |  | |  | |  | |  | |
| --- | --- | --- | --- | --- | --- | --- | --- | --- | --- |
| DV: pulled handle | | Coef. | | Std. Dev. | | Conf. Interval  2.5% | | Conf. Interval  97.5% | |
| Payoff_trialnum | | 9.61 | | 5.63 | | -5.82 | | 18.74 | |
| Payoff_is_0/1 | | -3.38 | | 41.93 | | -76.11 | | 86.62 | |
| Payoff_trialnum X Payoff_is_0/1 | | 0.57 | | 1.23 | | -2.23 | | 2.76 | |
| Constant | | -16.94 | | 95.76 | | -128.83 | | 162.32 | |
| **Level 2 estimates**  **Standard Deviations and Correlations between varying parameters** | | | | | | | | |  |
| DV: pulled handle | 1 | | 2 | | 3 | | 4 | |  |
| 1: Payoff_trialnum | 32.86 | |  | |  | |  | |  |
| 2: Payoff_is_0/1 | -0.22 | | 431.84 | |  | |  | |  |
| 3: Payoff_trialnum X Payoff_is_0/1 | -0.06 | | -0.15 | | 8.83 | |  | |  |
| 4: Constant | 0.21 | | -0.21 | | -0.06 | | 480.21 | |  |
