# Supplementary material for: Task Design Influences Prosociality in Captive Chimpanzees (Pan troglodytes)
Source: PLoS One. 2014 Sep 5;9(9):e103422. doi: 10.1371/journal.pone.0103422 (PMC4156467; doi:10.1371/journal.pone.0103422)
Supplement: Table S7 — Study 1, Knowledge Probe. Regression model for Figure S1, excluding varying estimates for each Actor. Similar model as in Table S6, but without allowing the parameters to vary across individuals. The interaction term is the most informative here, and the coefficient for this predictor is greater than zero. This was not obvious from the prior model in Table S5, because the parameter was allowed to vary across a small number of individuals (only four). (DOCX) [file pone.0103422.s009.docx]

**Table S7:** Similar model as in Table S6, but without allowing the parameters to vary across individuals.

The interaction term is the most informative here, and the coefficient for this predictor is greater than zero. This was not obvious from the prior model in Table S5, because the parameter was allowed to vary across a small number of individuals (only four).

| **Level 1 Estimates** |  |  |  |  |
| --- | --- | --- | --- | --- |
| DV: pulled handle | Coef. | Std. Dev. | Conf. Interval  2.5% | Conf. Interval  97.5% |
| Payoff_trialnum | -0.82 | 0.42 | -1.91 | -0.09 |
| Payoff_is_0/1 | 1.69 | 1.39 | -0.81 | 4.44 |
| Payoff_trialnum X Payoff_is_0/1 | 0.58 | 0.27 | 0.12 | 1.19 |
| Constant | -3.09 | 4.77 | -10.78 | 3.52 |
