# Supplementary material for: Task Design Influences Prosociality in Captive Chimpanzees (Pan troglodytes)
Source: PLoS One. 2014 Sep 5;9(9):e103422. doi: 10.1371/journal.pone.0103422 (PMC4156467; doi:10.1371/journal.pone.0103422)
Supplement: Table S8 — Study 2, Total number of observations contributed by each subject in Study 2, along with demographic data on subjects. Total N/Average N per animal, for Low dominance: 680/136. Total N/Average N per animal, for Medium dominance: 244/244. Total N/Average N per animal, for High dominance: 281/140.5. (DOCX) [file pone.0103422.s010.docx]

**Table S8:** Total number of observations contributed by each subject in Study 2, along with demographic data on subjects.

Total N / Average N per animal, for Low dominance: 680 / 136

Total N / Average N per animal, for Medium dominance: 244 / 244

Total N / Average N per animal, for High dominance: 281 / 140.5

| **Enclosure** | **Subject** | **Total *n* observations for this actor** | **Sex** | **Age (in years)** | **Birthplace,**  **Rearing history** | **Dominance level in group** |
| --- | --- | --- | --- | --- | --- | --- |
| 3 | 3012 | 237 | F | 45.46 | Wild,  unknown | Low |
| 3 | 3022 | 59 | F | 9.18 | Captive, mother raised | Low |
| 3 | 3032 | 133 | F | 46.46 | Wild,  unknown | High |
| 3 | 3052 | 29 | F | 20.31 | Captive, mother raised | Low |
| 4 | 4012 | 244 | F | 24.46 | Captive, mother raised | Medium |
| 5 | 5012 | 332 | F | 41.46 | Wild,  unknown | Low |
| 5 | 5022 | 148 | M | 39.46 | Wild,  unknown | High |
| 5 | 5032 | 23 | F | 47.46 | Wild,  unknown | Low |
