# Supplementary material for: Task Design Influences Prosociality in Captive Chimpanzees (Pan troglodytes)
Source: PLoS One. 2014 Sep 5;9(9):e103422. doi: 10.1371/journal.pone.0103422 (PMC4156467; doi:10.1371/journal.pone.0103422)
Supplement: Table S9 — Study 2, Regression models of the effect of Actor's Trial Number on actors' likelihood of choosing the prosocial option in Study 2, represented in Figure 5B . (DOCX) [file pone.0103422.s011.docx]

**Table S9:** Models of the effect of *Actor’s Trial Number* on actors’ choices of the prosocial option in Study 2, represented in Figure 5B.

| DV: Chose Prosocial Outcome  (For Control 1/1 coded as ‘1’) | Model 6 | Model 7 | Model 8 | Model 9 |
| --- | --- | --- | --- | --- |
|  | Rewards | Rewards  (food balanced) | No Rewards | Control |
|  | Coef. (SE) | Coef. (SE) | Coef. (SE) | Coef. (SE) |
| Actor’s Trial Number | < -.001 (.001) | .001 (.001) | < -.001 (.002) | .015 (.01) |
| Constant | .19 | -.06 | .31 | 3.16 |
| Random Effect | <.001 (.02) | .001 (.02) | .004 (.09) | .004 (.13) |
