# Supplementary material for: Task Design Influences Prosociality in Captive Chimpanzees (Pan troglodytes)
Source: PLoS One. 2014 Sep 5;9(9):e103422. doi: 10.1371/journal.pone.0103422 (PMC4156467; doi:10.1371/journal.pone.0103422)
Supplement: Table S10 — Study 2, Regression models of the effect of Actor's Trial Number on actors' likelihood of choosing neither handle in the 0/1 v. 0/0 condition of Study 2, represented in Figure 5B . (DOCX) [file pone.0103422.s012.docx]

**Table S10:** Models of the effect of *Actor’s Trial Number* on actors’ tendency to do nothing in the No Reward condition of Study 2, represented in Figure 5B.

| DV: Actor did nothing | Model 10 | Model 11 | Model 12 | Model 13 |
| --- | --- | --- | --- | --- |
|  | No Rewards | Rewards | Rewards  (food balanced) | Control |
|  | Coef. (SE) | Coef. (SE) | Coef. (SE) | Coef. (SE) |
| Actor’s Trial Number | .014 (.002) | .003 (.008) | .002 (.01) | .009 (.009) |
| Constant | -2.48 | -5.42 | -6.00 | -7.62 |
| Random Effect | 1.16 (.38) | .002 (.07) | .005 (.19) | 2.09 (1.99) |
